# Comparison of the Lag Screw Placements for the Treatment of Stable and Unstable Intertrochanteric Femoral Fractures regarding Trabecular Bone Failure

**DOI:** 10.1155/2016/5470798

**Published:** 2016-11-22

**Authors:** Talip Celik, Ibrahim Mutlu, Arif Ozkan, Yasin Kisioglu

**Affiliations:** ^1^Department of Biomedical Engineering, Kocaeli University, Umuttepe Campus, 41380 Kocaeli, Turkey; ^2^Department of Biomedical Engineering, Duzce University, Konuralp Campus, 81620 Düzce, Turkey

## Abstract

*Background. *In this study, the cut-out risk of Dynamic Hip Screw (DHS) was investigated in nine different positions of the lag screw for two fracture types by using Finite Element Analysis (FEA).* Methods. *Two types of fractures (31-A1.1 and A2.1 in AO classification) were generated in the femur model obtained from Computerized Tomography images. The DHS model was placed into the fractured femur model in nine different positions. Tip-Apex Distances were measured using SolidWorks. In FEA, the force applied to the femoral head was determined according to the maximum value being observed during walking.* Results. *The highest volume percentage exceeding the yield strength of trabecular bone was obtained in posterior-inferior region in both fracture types. The best placement region for the lag screw was found in the middle of both fracture types. There are compatible results between Tip-Apex Distances and the cut-out risk except for posterior-superior and superior region of 31-A2.1 fracture type.* Conclusion. *The position of the lag screw affects the risk of cut-out significantly. Also, Tip-Apex Distance is a good predictor of the cut-out risk. All in all, we can supposedly say that the density distribution of the trabecular bone is a more efficient factor compared to the positions of lag screw in the cut-out risk.

## 1. Introduction

Intertrochanteric Femoral Fractures (IFFs), also called proximal femoral fractures, usually occur between trochanter major and trochanter minor and these fractures are observed in old people in a quite common way, since they are prone to decline in bone density and strength due to aging. Apart from old people, young people can also experience these types of fractures as a result of sudden excessive force or stress. Stable and unstable fractures are the two types of proximal femoral fractures. In order to fix these two different types of fractures, either extramedullary implants such as Dynamic Hip Screw (DHS) or intramedullary implants such as gamma nail are used. Implant selection is vital for the treatment of stable and unstable trochanteric femoral fracture types. In stable IFF, extramedullary implants should be selected and intramedullary implants should be preferred for unstable IFF according to the recent studies [1, 2]. In addition to these studies, Parker and Handoll [3] who compared intramedullary and extramedullary implants for IFF concluded that DHS should still be considered as the gold standard device for stable and unstable IFF. Nevertheless, there are several common problems in the treatment of IFF with DHS such as implant failure and the cut-out of lag screw due to trabecular bone failure.

The mechanical role of the lag screw is to stabilize the fracture line preventing the slide and separation of fracture fragments. The force due to the body weight is transferred to distal femur via the DHS lag screw. On some occasions, high forces can cause the failure of DHS or trabecular bone. If the trabecular bone is damaged, a cut-out is seen. The cut-out can be defined as the scission of the implant from the inner region of the femoral head or a movement of the femoral head towards the varus direction. This leads to the retroversion of the lag screw and the femoral head. Multiple factors such as implant positions, bone quality, fracture types, and implant designs play a role in the cut-out risk [4]. Most of the clinical and biomechanical studies focused on the lag screw positions in the femoral head. However, which positions of the lag screw in the femoral head increase the cut-out risk and implant failure is still a controversial subject. Some researchers recommend the central placement [5], but others suggest the inferior and inferior posterior region in the lateral view [3, 6, 7]. Some authors believe that the cut-out risk in posterior (P) region is lower compared to the other region [4]. There is no unanimous agreement on the ideal position of the lag screw in the femoral head. Besides these recommendations, implant failure was also reported in DHS fixation depending on lag screw position [8]. A method has been developed by Baumgaertner et al. [9], called Tip-Apex Distance (TAD), the length of the distance from the tip of the lag screw to the apex of the femoral head on an anteroposterior and lateral radiograph, to estimate the cut-out risk. This method has been used as a reliable method in most clinical practices [1, 5, 10–12]. Nonetheless, according to a recent study, TAD is not an accurate predictor for the cut-out [6]. Another factor of the cut-out risk and implant failure is the fracture type of femur trochanteric region. Stable and unstable IFFs affect the load transfer from hip to distal femur. Therefore, the implant selection and position in the femoral head are of paramount importance for different IFF types in terms of the cut-out of the lag screw and implant failure [13].

In this study, the effects of three factors (lag screw positions, fracture types, and TAD) in the cut-out risk were evaluated using Finite Element Analysis (FEA) in a patient-specific femur. The aim of the FEA study was to assess how the different positions of the lag screw and fracture types can influence the risk of cut-out systematically.

## 2. Materials and Methods

### 2.1. Model Development

**Figure 1:**
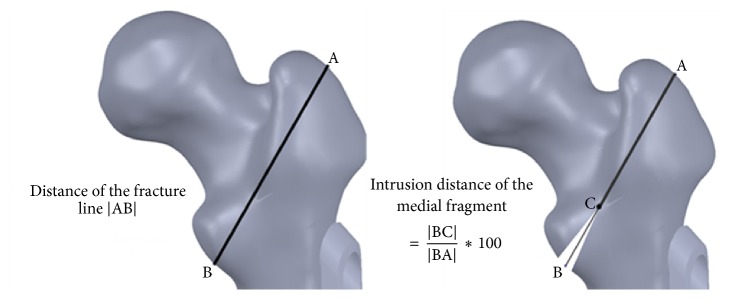
31-A1.1 and 31-A2.1 Muller AO classification and intrusion distance of the medial fragment is defined as the ratio of the length of the wedge to the length of the fracture line in the AP view.

**Figure 2:**
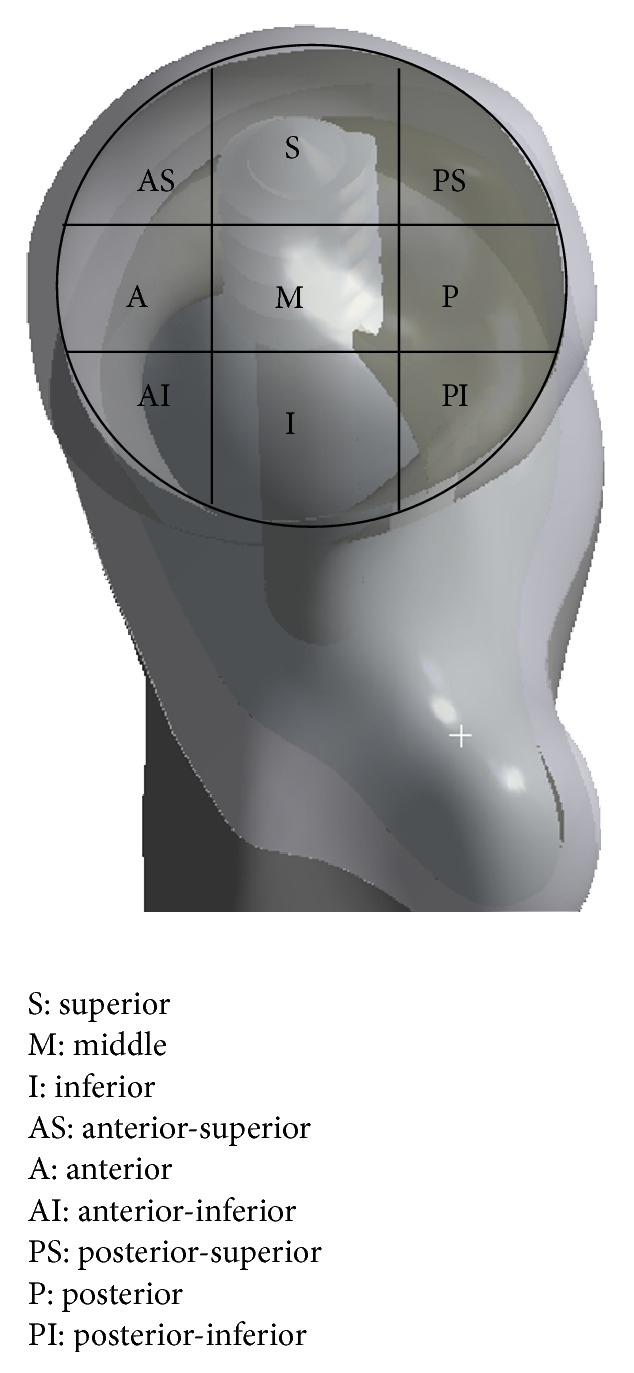
The femur head was divided into nine different positions in accordance with clinical practice.

**Figure 3:**
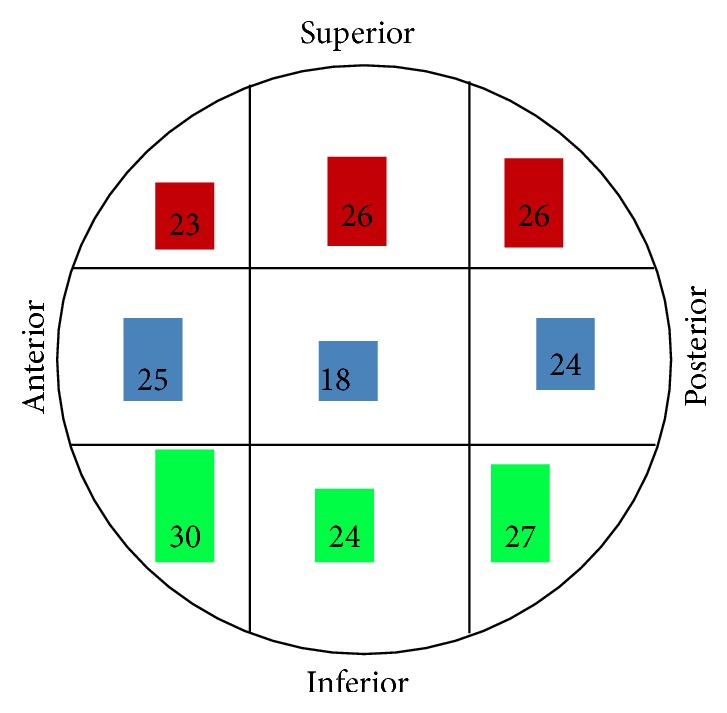
TAD values (in mm) of all DHS regions on the femoral head. Numbers in red, blue, and green indicate superior, middle, and inferior region, respectively.

 3D femur cortical and trabecular models were modelled via computerized tomography (CT) images obtained from a male patient aged 57 using a Toshiba Aquilion CT scanner in the Department of Radiology of the Medicine Faculty at the University of Kocaeli. CT images consist of parallel layers having a pixel size of 0.774 × 0.774 mm at the lateral position and a voxel resolution of 473 × 473 × 235. 1841-layer shooting was carried out to develop the model. The images were recorded in the Digital Imaging and Communications in Medicine (DICOM) format. These images were then transferred to the MIMICS 12.1 (Materialise, Leuven, Belgium) 3D image-processing software. The surface errors (spike, intersection, etc.) of the models of femur cortical and femur trabecular bones were corrected with the help of Geomagic Studio 10 software (Raindrop Inc., USA). After the correction of the surface roughness of the model, 3D smooth solid models were developed and imported into SolidWorks program (Dassault Systèmes SolidWorks Corp., USA) in IGES format. In these models, two- and three-part trochanteric fractures were formed as 31-A1.1 (stable fracture type) and 31-A2.1 (unstable fracture type) in the Muller AO classification [13] accompanied with and without medial support at the level of the lesser trochanter in SolidWorks program. The angle of the fracture line with the femoral anatomic axis was assumed to be 30° and the proportion of the intrusion distance of the medial fragment to the distance of the fracture complex was assumed to be 30% that is mostly encountered [15] (Figure 1). The geometrical dimensions of the DHS with a 130° four-hole standard barrel plate were obtained from the implant manufacturer catalogue [TIPSAN Co. Inc.]. The femoral head was schematically divided into nine different positions as shown in Figure 2. The models of DHS and fractured femur were combined with different lag screw positions in accordance with clinical practice. TAD values were measured in both AP and lateral views in SolidWorks program as suggested by Baumgaertner et al. [9] and were illustrated as a gauge bar in the femoral head (Figure 3). Finally, the femur models with DHS implants positioned as mentioned above were imported into ANSYS Workbench software (ANSYS Inc., Canonsburg, PA) in IGES file format for FEA.

### 2.2. Properties of Material Mesh and Contact Assignments

It was assumed that the material properties of the bones and DHS models are linear elastic and isotropic. The material of DHS was considered to be made of 316L stainless steel which is commonly utilized in the treatment of IFF. The material property values that were obtained from the literature were determined as shown in Table 1 [14]. Mesh convergence was tested by refining the element size from 6 to 3 at 1 mm interval on the femur and 4 to 1 at 0.5 mm interval on the DHS plate. The most suitable element size for the optimum results was determined as 4 mm and 1.5 mm for the whole femur and DHS plate, respectively. The element types of Solid 186 (hexahedron-dominant) and Solid 187 (tetrahedron) were used in the whole finite element model. In FEA, several mesh sizes for the additional refinement were defined at some critical locations such as the screw threads and the corner of the DHS model in order to get convergence. The number of elements and nodes changed from 65.000 and 150.000 to 75.000 and 245.000, in a successive way. The interactions of all contact surfaces were presumed as a frictional contact except the interactions between trabecular and cortical bone. Friction coefficients were defined as 0.42 for the interactions between the bone and the implant [16], 0.2 for the interactions between the implant and the fragment of the implant itself [17], and 0.46 for the interactions of the fragments of the fractured bone [18].

### 2.3. Boundary Conditions

**Figure 4:**
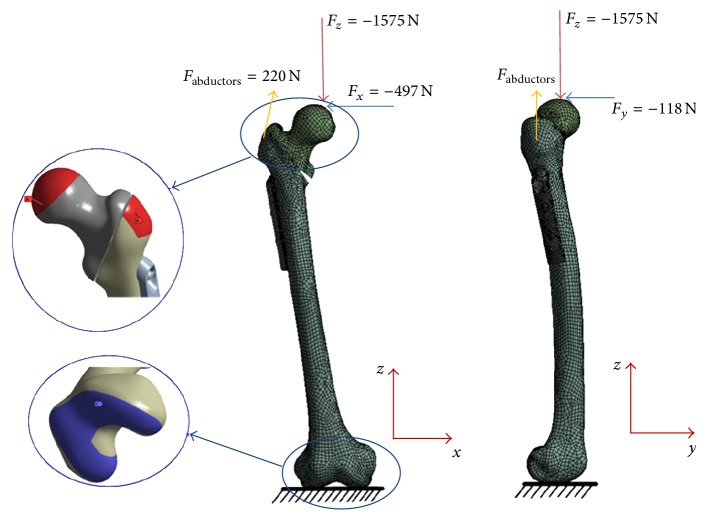
The loading and boundary conditions for this study. The forces applied to the femoral head surface and the femur model constrained in all directions of distal femur knee joint region.

The fractured femur models fixed with DHS were subjected to a static load obtained from the literature in accordance with the value reported for a person walking at a normal speed [19]. The coordinate system for the femur was defined based on the definition by Bergmann et al. [19]. Considering body weight, the maximum forces resulting from walking were applied to the femoral head surfaces in ANSYS Workbench for *x*-*y*-*z* force vectors, as shown in Figure 4. The force of the abductor muscle was applied as presented by Duda et al. [20]. The distal ends of the fractured femur models were constrained taking into consideration the contact surface of the knee joint as shown in Figure 4.

### 2.4. Evaluation Method for the Cut-Out Risk

The compressive strain criterion was selected to predict the cut-out risk of the femoral head models in the trabecular bone according to Schileo et al. [21]. Expected femur trabecular bone failure is supposed to occur when the strain level exceeds the trabecular bone yield strain equaling to 1% of the compressive strain of the trabecular bone [22, 23]. The volume percentages of the trabecular bone exceeding the yield strength of the compressive strain for each position were calculated and compared to each other. The best lag screw location was determined according to the amount of minimum volume percentage.

## 3. Results

### 3.1. Strain Analysis

**Figure 5:**
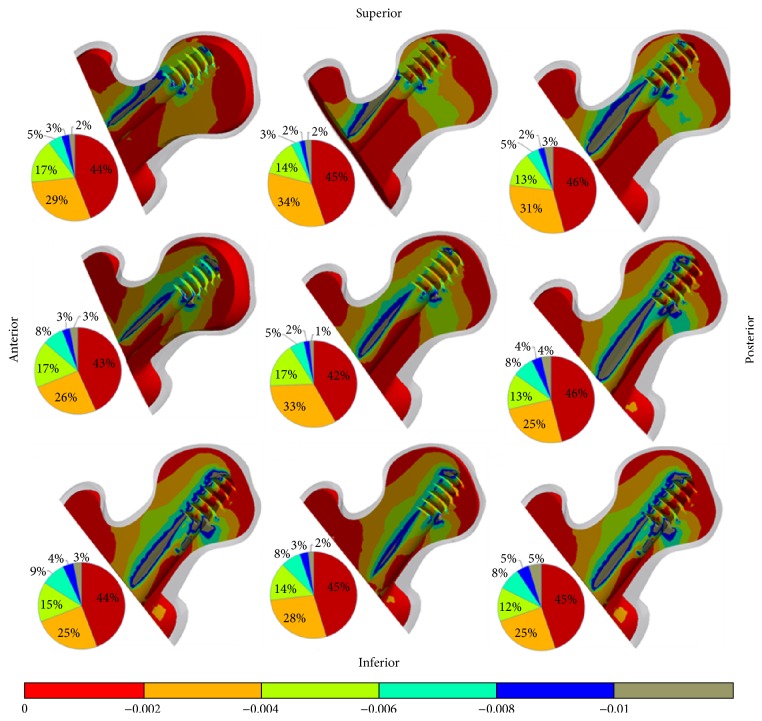
The anterior-posterior sectional views of the compressive strain distributions of the femur trabecular heads (31-A1.1 fracture type) in nine DHS positions and graphical percentages of the strain values according to the gauge bar. The regions having strain values at below 1% are under a higher risk of trabecular bone failure.

**Figure 6:**
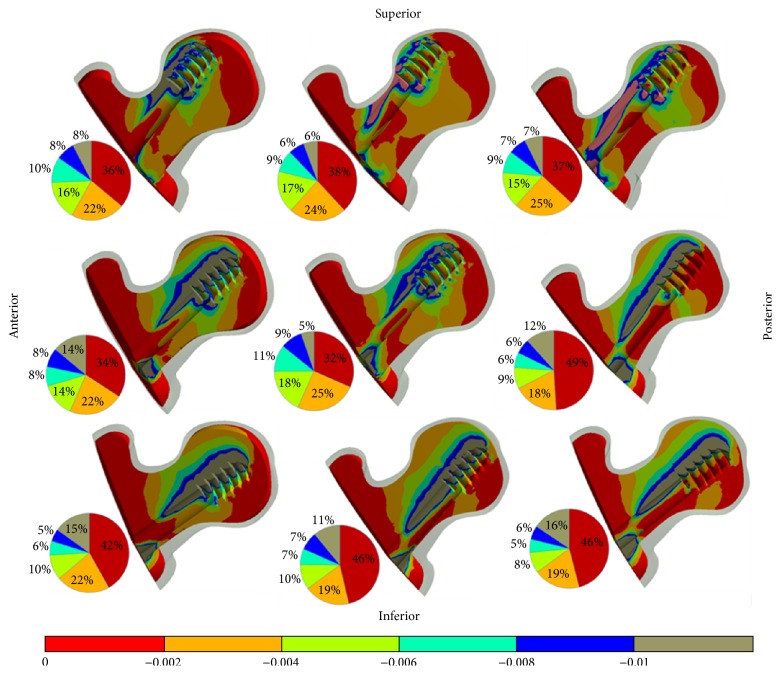
The anterior-posterior sectional views of the compressive strain distributions of the femur trabecular heads (31-A2.1 fracture type) in nine models and graphical percentages of the strain values according to the gauge bar. The regions having strains values at below 1% are under a higher risk of trabecular bone failure.

The contour plots in Figures 5 and 6 illustrate the minimum principal strain (compressive) results in a cross section of the trabecular femur head for nine different models with both fracture types. Furthermore, the volume percentages of the compressive strain level on the trabecular femur are shown as a pie chart in Figures 5 and 6. The gauge bar in Figures 5 and 6 indicating strain levels was divided by strain bands. The maximum value in the gauge bar was accepted as 1% of the compressive strain of the trabecular bone. Based upon the compressive strain criterion, posterior regions (P, PI) of 31-A1.1 fracture type models had the largest failure regions on the trabecular bone which is close to DHS-femur neck region as shown in Figure 5. The lowest cut-out risk was specified in the middle region with reference to the trabecular bone failure criterion. The AS, S, and I regions had a lower cut-out risk compared to the PI and P regions in a comparison with the percentages surmounting the compressive strain at a rate of 1%.

As expected, higher compressive strain values were predicted for the 31-A2.1 compared to 31-A1.1 fracture types owing to the load transfer pathway of the femur. The values of all A2.1 fractured femur models surpassed the value of the compressive strain at 1%. Accordingly, all regions for both fracture types were at the risk of a cut-out in defined loads and boundary conditions. The values in excess of the strain values at 1% were detected at the upper side region of the lag screw and at the intersection region between the lag screw and fracture surfaces for all models. Pertaining to the results, as the most suitable region for the cut-out risk, the middle placement of the lag screw was determined with reference to the yield strain criterion of the trabecular bone. The higher cut-out risk regions were the PI, AI, and A regions as shown in Figure 6.

### 3.2. TAD Analysis

All TAD values as to the lag screw positions were fewer than 30 mm except the TAD value of AI position having the maximum values at 30 mm as shown in Figure 3. The results demonstrated that the PI and AI regions had the higher risk and also the higher TAD values. Although the PS and S regions had higher TAD values compared to the AS, A, P, and I regions, the volume percentages of the trabecular failure in the PS and S regions were less than in AS, A, P, and I regions. Hence, the results of TAD proved incompatible in the regions of PS and S in A2.1 fracture type.

## 4. Discussion

The cut-out risk of the lag screw in two IFF types can be estimated by utilizing the technique of the FE simulations. The clinical treatments of the femur fractures can be numerically assessed. Biomechanical studies on the lag screw positions usually evaluate only the positions of S, M, and I regions [7, 24] as shown in Figure 3 contrary to many clinical studies [5, 10–12]. There is no biomechanical or clinical study about how fracture types affect the cut-out risk in different lag screw positions according to our literature search. In this study, the effects of the varieties of the lag screw positions observed in clinical practices on the cut-out risk were evaluated using FE method in two types of the femur trochanteric fractures.

The forces of the abductor muscle were applied in FEA and the other muscles were ignored in this study. Eberle et al. reported that the muscle forces had no significant effect on the strains of the intramedullary nail used for the treatment of femur trochanteric fractures [14]. Besides, Konstantinidis et al. concluded that the muscle forces have little effect on the fracture displacement for trochanteric fractures [25]. Indeed, particularly the force of the abductor muscle has a fundamental effect on the strain values of the femur [26, 27]. In the light of these studies, we arrived at a decision that only the force of the abductor muscle should be added to FEA.

The critical strain value was exceeded in all positions of both fracture types. Comparing the volume percentages of the strain between each other in all positions, we found that the lag screw placements of P regions (P, P-I, and P-S) and I regions (A-I, I, and P-I) of the femoral head increased the failure risk of the trabecular bone. In contrast, the placement of the lag screw in the middle region leads to a lower risk compared to other placements. The results seem inconsistent with other studies [3, 5–8, 28]. These incompatible results originated from several reasons such as the type of fixation [3], femur fracture type models [5, 6], boundary conditions [6–13, 15], and different TAD values [3, 5–8, 28]. Another reason for incompatibility between this study and clinical studies [8, 28] is the complex structures of the trabecular bone. In reality, the material structure of the proximal part of the trabecular bone is very complex and it is very difficult to represent this complex structure of the bone material in FEA. It was assumed that the femur consisted of isotropic material and homogeneous trabecular bone density. Upon taking a glance at studies about bone density, we realized that the central region of the femoral head had the strongest trabecular bone, whereas the superior regions of the head had the weakest trabecular bone [29, 30]. Under these circumstances, the cut-out risk may increase at the superior region and decrease at the center and inferior regions. As to these factors, even though the superior regions seem to be safer than the other regions except the region of M in our study, the possibility of the cut-out in superior regions is higher than expected. On the other hand, the possibility of the cut-out at the AI, I, PI, A, and P regions is lower than expected. Fundamentally, the assumptions of the homogeneous isotropic properties for the trabecular bone simplify the models and thus weaken the conclusions. Therefore, with our study the fact that the density distribution of the trabecular bone is a more efficient factor compared to the positions of the lag screw in the cut-out risk considering the clinical study outcomes [5, 6, 8] can be presumably stated. Further studies can be conducted about how the elasticity of different trabecular bone regions affects the strain results of the patient's femoral head.

The highest compressive region was found at the upper region of the lag screw threads on the trabecular bone as illustrated in Figures 5 and 6. The cut-out can begin from this region. However, according to the recent study [6], a critical region was determined in different places most of which were near the intersection between the fracture line and the lag screw unlike our finding. The mentioned region has a secondary importance for our study. The main reason for this discrepancy can be originated from modelling taking into consideration the bone density of the femoral head and the TAD values. In addition, the loads are transferred to the knee via the lag screw in A2.1 fracture type. Nonetheless, the transferring path of loads in A1.1 fracture type is via both the lag screw and the fracture surfaces with the medial support. While the load is transferred to the distal femur, the fragments of the femoral head are enforced to be separated from each other in the superior region of the fracture line. Conversely, the fracture surfaces in the inferior region of the fracture line are pressed against each other. Thus, the critical regions of the trabecular bone are observed at the tip of the lag screw and at the intersection region between the lag screw and the fracture line.

Lag screw placement plays an important role in transferring load. TAD depends on the placement of the lag screw into the femoral head and thereby affects the strain on the trabecular bone. A significant number of clinical studies have been carried out for the relation between TAD and the cut-out. These studies regard TAD as the best predictor for the cut-out risk [5, 9–12, 30]. Nevertheless, it is not an accurate predictor in that it cannot reflect the inhomogeneous distribution of the bone in femoral head regions [9]. If just TAD is used to determine risk, it may cause a higher risk of failure due to the placement of superior regions [29]. Based on our results, the risk of the cut-out is predicted by TAD except the S and PS placements in 31A2.1 fracture type regarding the volume percentages of strain exceeding the yield point. One reason for not being predicted in S and PS placements can be explained by the fact that the volume of the trabecular bone at the tip of the lag screw is less than that of other regions. Other reasons can be explained by the ignorance of the distribution of the bone density and the selection of volume percentages regardless of the maximum strain values as an evaluation method. Consequently, there is a compatible relation between the cut-out risk and TAD values as the other numerical and clinical studies have found [5, 6, 8, 10–12, 28].

There are some other limitations of this FE study. One of them is the material properties of the bone tissues. These are usually assumed to be the linear, homogeneous, and isotropic material properties in many FEA studies [6, 24] such as in this study. However, these tissues have anisotropic and heterogeneous material properties [18]. One other limitation is that the behaviour of muscle and tendon tissues surrounding the fracture location was not included. These tissues may affect the loads and fracture locations. In our opinion, these tissues effects are lower and limited. Another limitation of the study is that the only normal walking loading case which did not create the worst loading case for the models was used. The other loading case such as stair climbing may give different information. Further investigation on the different load effects might be studied in the future. The other limitation is that the femur-implant interfaces were assumed to be ideally contacted with each other. In reality, contact ratio is changeable from patient to patient according to application of surgeon and bone porosity. These variables are difficult to simulate with the FEA. Therefore, the results from this study might be interpreted under ideally contacted conditions. The validation of the FE model is crucial to take accurate results. In this study, experimental validation was not made. Instead, validity of the FE model was accepted by making the convergence study. Furthermore, the resulting comparisons of the eighteen models give the idea that the results are accurate.

The FEA results showed that femur trochanteric fracture types are crucial for the treatment of the femur fractures. In other words, fracture types affect the cut-out risk in different lag screw positions. Some obvious distinctions were recorded between the stable (31-A1.1) and unstable (31-A2.1) fracture types in terms of the minimum principal strain distributions. The unstable fracture increasing the strain levels of the femoral head caused a higher cut-out risk of the lag screw. For this reason, there is a possibility of the trabecular bone yielding at the tip of the lag screw in the unstable fracture models.

## 5. Conclusions

The bone density and the location of the lag screw at the femoral head are fundamental factors for the cut-out risk. In addition, the fracture type of IFF affects the cut-out risk. Furthermore, the method of TAD used for the determination of the cut-out risk in clinical practices also proves to be useful as a predictor in our study. All in all, we can supposedly say that the density distribution of the trabecular bone is a more efficient factor compared to the positions of the lag screw in the cut-out risk.

## Figures and Tables

**Table 1:** Material properties of femur cortical and trabecular bone and DHS implant [14].

	Young's modulus (MPa)	Poisson's ratio	Density (kg/m^3^)
316L stainless steel (DHS)	193000	0.3	8000
Cortical bone	15000	0.3	1900
Trabecular bone	1050	0.3	700
